# Autistic adults show preserved normalisation of sensory responses in gaze processing

**DOI:** 10.1016/j.cortex.2018.02.005

**Published:** 2018-06

**Authors:** Colin J. Palmer, Rebecca P. Lawson, Shravanti Shankar, Colin W.G. Clifford, Geraint Rees

**Affiliations:** a School of Psychology, UNSW Sydney, NSW, Australia; b Institute of Cognitive Neuroscience, UCL, London, UK; c Wellcome Trust Centre for Neuroscience, UCL, London, UK; d Department of Psychology, University of Cambridge, Cambridge, UK

**Keywords:** Gaze perception, Adaptation, Divisive normalisation, Autism, Neural computation

## Abstract

Progress in our understanding of autism spectrum disorder (ASD) has recently been sought by characterising how systematic differences in canonical neural computations employed across the sensory cortex might contribute to clinical symptoms in diverse sensory, cognitive, and social domains. A key proposal is that ASD is characterised by reduced *divisive normalisation* of sensory responses. This provides a bridge between genetic and molecular evidence for an increased ratio of cortical excitation to inhibition in ASD and the functional characteristics of sensory coding that are relevant for understanding perception and behaviour. Here we tested this hypothesis in the context of gaze processing (i.e., the perception of other people's direction of gaze), a domain with direct relevance to the core diagnostic features of ASD. We show that reduced divisive normalisation in gaze processing is associated with specific predictions regarding the psychophysical effects of sensory adaptation to gaze direction, and test these predictions in adults with ASD. We report compelling evidence that both divisive normalisation and sensory adaptation occur robustly in adults with ASD in the context of gaze processing. These results have important theoretical implications for defining the types of divisive computations that are likely to be intact or compromised in this condition (e.g., relating to local *vs* distal control of cortical gain). These results are also a strong testament to the typical sensory coding of gaze direction in ASD, despite the atypical responses to others' gaze that are a hallmark feature of this diagnosis.

## 1 Introduction

Autism spectrum disorder (ASD) is a heterogeneous developmental condition, characterised by differences in social interaction, a strong preference for routine, repetitive motor behaviours, and sensory sensitivities (American Psychiatric Association, 2013, Lai et al., 2014). ASD has a strong yet highly complex genetic basis (Geschwind & State, 2015), and there is currently no explanation of the condition that bridges biological, cognitive, and behavioural levels of description. Recently, progress has been sought by drawing on general *computational theories of brain function* to characterise how systematic differences in the processing of sensory information may contribute to the sensory and social symptoms of ASD (e.g., Lawson et al., 2014, Palmer et al., 2017, Rosenberg et al., 2015, Van de Cruys et al., 2014). These theories highlight the *control of cortical gain* as a computationally-important neural mechanism that a variety of genetic and molecular differences might converge on.

There is genetic and molecular evidence for an increased ratio of cortical excitation to inhibition in ASD (e.g., Rubenstein and Merzenich, 2003, Yizhar et al., 2011), and computationally, this can be related to the *divisive normalization* of sensory responses (Rosenberg et al., 2015). Divisive normalization occurs when the responses of a sensory neuron are not only driven by stimuli that excite it, but also modulated by the responses of local, functionally-related cell populations (e.g., those with adjacent spatial receptive fields). This is a form of neural gain control that may be instantiated by lateral inhibitory connections in sensory areas of the cortex. It is now well-established that this computation is employed in a widespread manner across sensory systems (Carandini & Heeger, 2012), playing an essential role in maintaining a sensory code that is robust to extraneous, context-dependent variation in neural firing.

Correspondingly, a key proposal is that symptoms in ASD, across sensory, cognitive, and social domains, reflect a widespread *reduction* of divisive normalisation in neural processing (Rosenberg et al., 2015). This hypothesis is attractive in its potential to link our expanding knowledge of the complex biological underpinnings of this condition to functional characteristics of sensory coding, and thereby perception and behaviour. Initial support for this idea comes from simulation analyses that demonstrate that certain low-level visual characteristics in ASD (e.g., weak visual spatial suppression) can feasibly arise through reduced normalisation of sensory responses in primary visual cortex (Rosenberg et al., 2015). Rosenberg and colleagues also argue that the notion of reduced normalisation computations, if a systemic feature of neural processing in ASD, can help to make sense of experimental data across a variety of domains, including local versus global processing, multisensory integration, and decision-making. However, the proposal as a whole largely remains to be tested, including how the proposed differences in sensory processing contribute to the behaviours defining the diagnostic criteria.

In the social domain, recent research has examined the role of divisive normalisation in the sensory coding of others' direction of gaze (Palmer and Clifford, 2017a, Palmer and Clifford, 2017b). This has revealed a distinct psychophysical signature of normalisation in neurotypical (NT) individuals, reflected in the fine-grained effects of *sensory adaptation* on subsequent perception of gaze direction. Sensory adaptation occurs when prolonged viewing of a specific direction of gaze (e.g., far leftwards averted gaze) causes a repulsive aftereffect such that subsequently presented faces are seen as looking more rightwards than their veridical direction of gaze. This phenomenon is thought to reflect targeted habituation of stimulus-selective sensory channels, and can be used to probe the underlying sensory coding of perceptual properties like gaze direction (Suzuki, 2005). The adaptive sensory coding of gaze direction is linked to cortical function in higher visual areas, namely anterior superior temporal sulcus (Calder et al., 2007, Carlin and Calder, 2013).

It is appealing to examine the function of divisive normalisation in ASD in the context of gaze perception, because atypical gaze-based behaviours are a cardinal diagnostic feature of ASD. This includes, for instance, a reduced tendency to seek mutual gaze when interacting with others, in both childhood and adulthood. Experimental research in ASD has shown differences in how attention is cued on the basis of others' gaze direction (Frischen, Bayliss, & Tipper, 2007), reduced salience of direct gaze (Senju, 2013), and subtle differences in the sensory coding of others' gaze direction, namely a reduced influence of recent sensory history on current perception (Lawson, Aylward, et al., 2017, Pellicano et al., 2013). Prominent social-cognitive theories also emphasise the role of eye gaze processing in our ability to make inferences about other people's mental states, issues which are commonly thought to be a core driver of social difficulties in ASD (Baron-Cohen, 1997, Lai et al., 2014).

Here we present a computational simulation analysis demonstrating that reduced divisive normalisation in the context of gaze perception is associated with distinct predictions regarding the psychophysical effects of sensory adaptation to gaze direction. Correspondingly, we compare sensory adaptation to gaze direction between adults with ASD and NT adults. This allows us to (1) empirically test the proposal that ASD is characterised by reduced divisive normalisation of sensory responses (Rosenberg et al., 2015), in a domain pertinent to the social symptoms of this condition, and (2) probe for differences more generally in the functional mechanisms that underlie sensory processing in the cortex, namely the adaptive coding of others' gaze direction across gaze-selective sensory channels. We find compelling evidence that the adaptive coding of others' gaze direction occurs as robustly in adults with ASD as in NT controls, including in the divisive normalisation of sensory responses. These results further our understanding of how information about others' gaze is processed in ASD, and help to adjudicate between recent computational accounts of this condition that emphasise problems in local versus distal gain control in sensory function (Lawson, Friston, & Rees, 2015).

## 2 Material and methods

### 2.1 Simulation of reduced normalisation in the sensory coding of gaze direction

#### 2.1.1 Computational model of perceived gaze direction

Electrophysiological studies in macaque monkeys have identified individual cells in temporal cortex sensitive to the gaze direction of a seen face (Perrett et al., 1992, Perrett et al., 1985). Psychophysical and functional neuroimaging research in humans similarly indicate that perceived gaze direction is coded across distinct neuronal populations tuned to different directions of gaze (e.g., leftwards *vs* rightwards gaze) (Calder et al., 2008, Calder et al., 2007, Jenkins et al., 2006, Seyama and Nagayama, 2006). Recently, we developed a computational model of how information is combined across a set of direction-specific sensory channels to encode the perceived direction of gaze (Palmer & Clifford, 2017a). We found that different functional architectures made distinct predictions regarding the effects of sensory adaptation on the subsequent perception of gaze direction. Thus, in this previous work, we compared the predictions of these different models to the effects of adaptation to gaze direction observed empirically. Our results indicated that the effects of sensory adaptation were explained well by a model in which perceived gaze direction was coded in terms of the relative activation across three sensory channels tuned broadly to leftwards gaze, direct gaze, and rightwards gaze, and in which the encoded gaze direction was normalised to the summed activation across these sensory channels (described further below). In the present section, we simulate the effects of *reduced normalisation* within this model of gaze coding, and find that different degrees of normalisation are associated with different predictions regarding the psychophysical effects of adaptation to averted gaze.

**Fig. 1:**
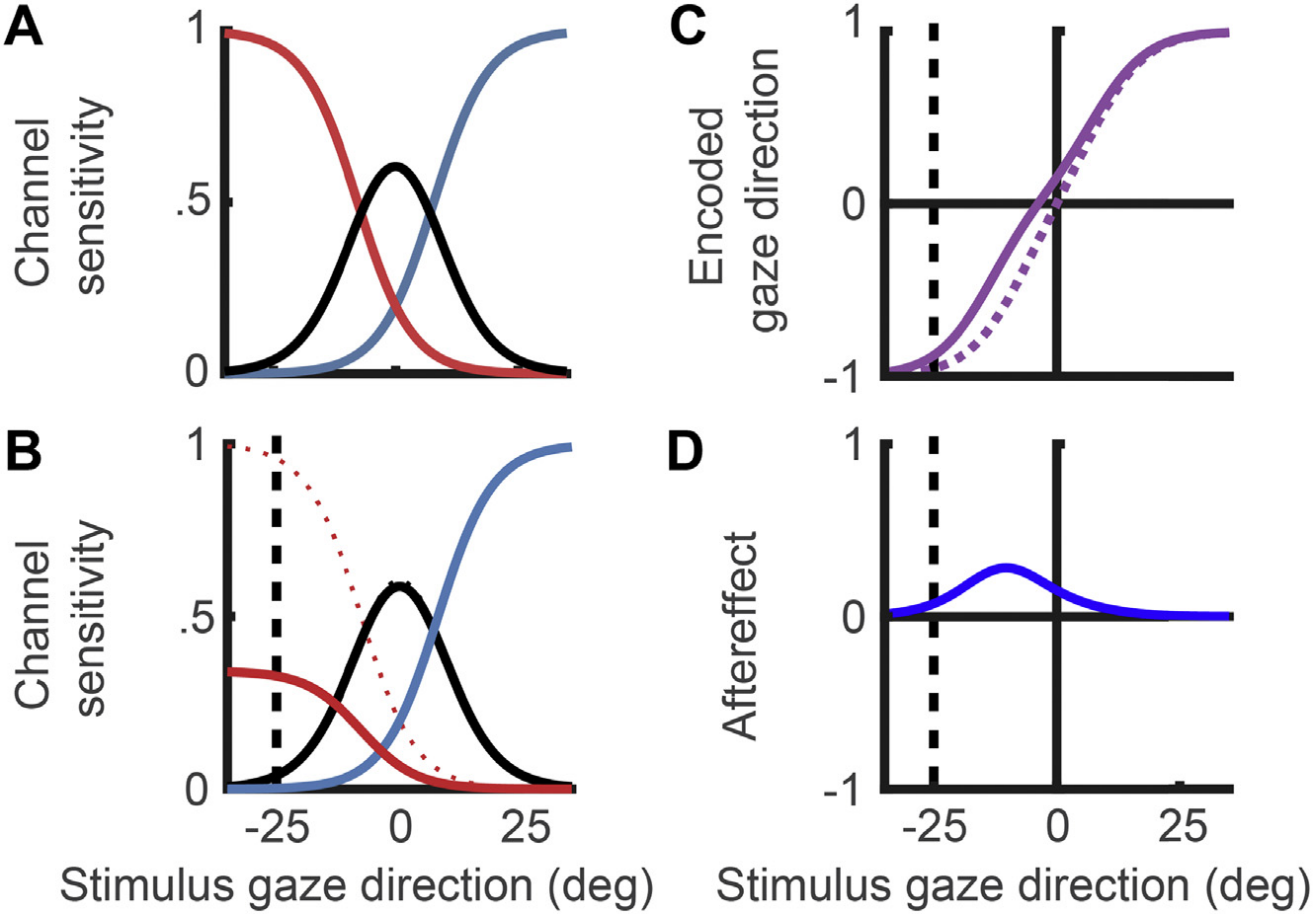
Modelling sensory coding and sensory adaptation in the context of gaze perception. **(A)** A model of the coding of horizontal gaze direction, with three sensory channels tuned broadly to leftwards, direct, and rightwards gaze directions, respectively. **(B)** Adaptation to 25° leftwards gaze is modelled as a reduction in the gain on channel sensitivities proportional to how strongly each channel is engaged by this stimulus. The direction of the adapter is shown by the vertical dotted line. **(C)** The encoded gaze direction computed from the relative activation across the three sensory channels, before adaptation (dotted line) and after adaptation (solid line). **(D)** The predicted change in perceived gaze direction induced by adaptation (i.e., the perceptual aftereffect), computed by comparing the encoded gaze direction within the model before and after adaptation.

A three-channel model of perceived gaze direction is illustrated in Fig. 1A, which depicts the sensitivity of each channel as a function of the stimulus gaze direction. The sensitivity of the leftwards channel (shown in red), *L*, is described by a logistic function, as follows, where *d* is the stimulus gaze direction, *g* sets the gaze direction at which the channel sensitivity is half maximum, and *s* sets the steepness of the slope:

$$L\left(d\right)=\frac{1}{1+e^{+\left(\frac{d+g}{s}\right)}}.$$

The sensitivity of the channel tuned to rightwards gaze (shown in blue), *R*, is set as a mirror image of *L*. The sensitivity of the channel tuned to direct gaze (shown in black), *C*, is set such that the three channels sum to 1. Thus, defining the channel sensitivities in the unadapted state requires just two parameters (*g* and *s*). Plausible values for these parameters were obtained by fitting the model to a set of gaze adaptation data reported in Calder et al. (2008). The fit of the model to these previous data is described in Palmer and Clifford (2017a). The best fitting parameters were *g* = 7.78° and *s* = 6.40°.

The effects of adaptation to a given direction of gaze were modelled as a reduction in channel sensitivity proportional to how strongly the channel was engaged by the adapting stimulus, as follows:

$$R_{A}\left(d\right)=\left(1-\alpha R_{0}\left(d_{A}\right)\right)*R_{0}\left(d\right),$$

$$L_{A}\left(d\right)=\left(1-\alpha L_{0}\left(d_{A}\right)\right)*L_{0}\left(d\right),$$

$$C_{A}\left(d\right)=\left(1-\alpha C_{0}\left(d_{A}\right)\right)*\;C_{0}\left(d\right).$$

where α determines the degree of adaptation and the subscripts 0 and A denote pre- and post-adaptation responses, respectively. This is illustrated in Fig. 1B. As above, a plausible value for α was obtained by fitting the model to data from Calder et al. (2008), as described in Palmer and Clifford (2017a). The best fitting parameter was α = .69.

#### 2.1.2 Simulating reduced normalisation within this model

The model of encoded gaze direction that we have previously found to fit well with perceptual aftereffects observed in a neurotypical population takes the form as follows, where *M* is the encoded direction of gaze:

$$M\left(d\right)=\;\frac{R\left(d\right)-L\left(d\right)}{R\left(d\right)+L\left(d\right)+C\left(d\right)}\;.$$

In this equation, the encoded gaze direction is expressed as the difference in activity between leftwards and rightwards sensory channels, normalised to the summed activity across sensory channels. Normalisation is important to sensory coding by making the encoded parameter (e.g., direction of gaze) robust to variations in extraneous factors that might otherwise influence neural responses, such as stimulus contrast. Here, normalisation to the summed activity across gaze-selective sensory channels makes the encoded gaze direction robust to factors that affect the activity of these channels equally. Using this model of encoded gaze direction together with the modelled effects of sensory adaptation on channel responses described in the previous section, we can compute the encoded gaze direction for a set of stimuli before and after adaptation, and thus derive predictions of the shift in perceived gaze direction induced by adaptation (i.e., the sensory aftereffect; illustrated in Fig. 1C and D).

To simulate the effects of reducing the extent of normalisation on perceived gaze direction, we included a further term, *w*, as follows:

$$M\left(d\right)=\;\frac{R\left(d\right)-L\left(d\right)}{w+\left(1-w\right)\left(R\left(d\right)+L\left(d\right)+C\left(d\right)\right)}\;.$$

The value of *w* sets the extent to which the encoded gaze direction is normalised to the summed activity across sensory channels. When *w* = 0, the encoded gaze direction is fully normalised to the summed activity across sensory channels. When *w* = 1, there is no normalisation of the encoded gaze direction.

**Fig. 2:**
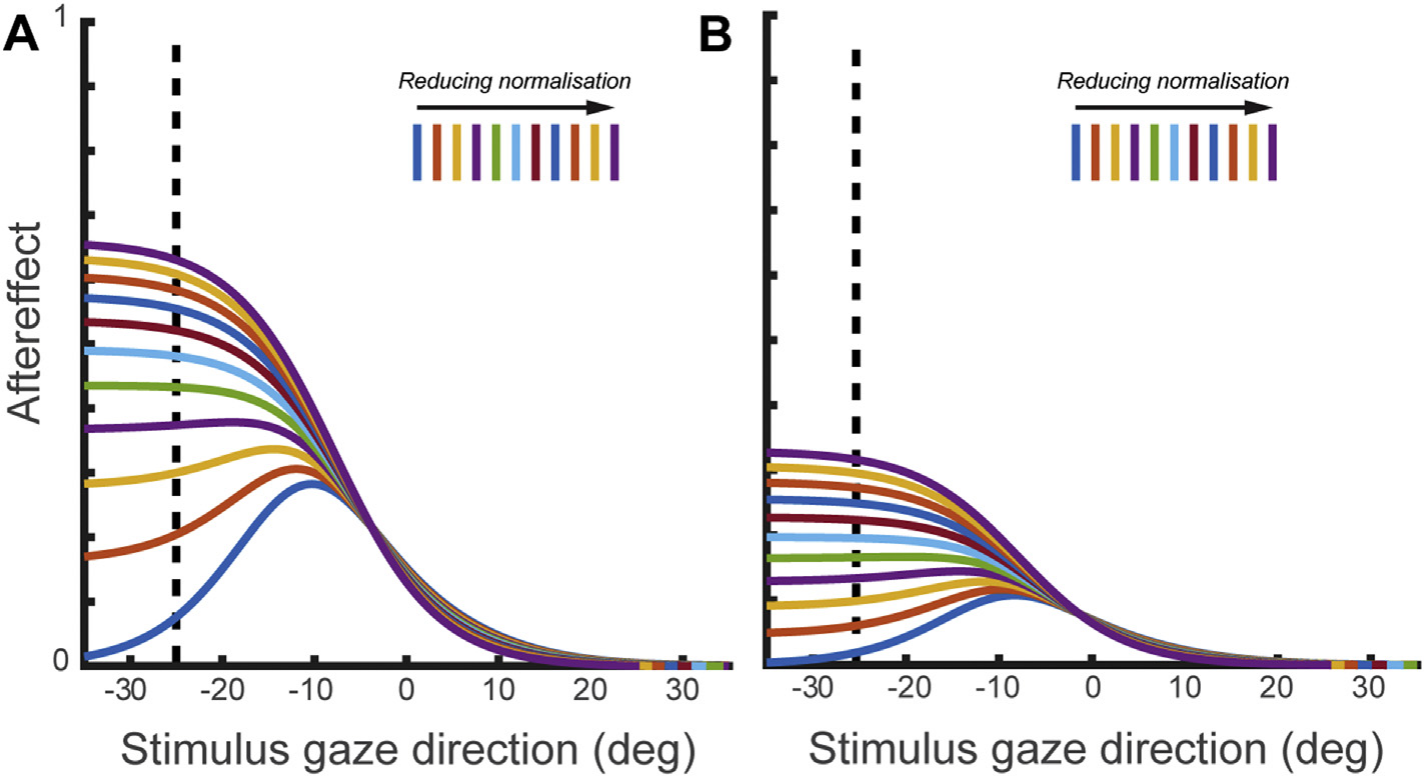
Simulating the effect of reduced normalisation of sensory responses to gaze direction on the perceptual effects of sensory adaptation. **(A)** Predicted effects of adaptation to 25° leftwards gaze across models with different degrees of normalisation. The effects of adaptation are expressed as perceptual aftereffects, i.e., the change in perceived gaze direction following adaptation compared to a pre-adaptation test of gaze perception. **(B)** Same as (A) but with 50% reduced strength of adaptation.

We simulated the effect that reducing normalisation would have on the perceptual effects of adaptation to gaze direction. Fig. 2A plots the predicted effects of adaptation to 25° leftwards gaze on the subsequent perception of gaze direction, as *w* is varied between 0 (full normalisation) and 1 (no normalisation) in increments of .1.

Individuals with ASD show reduced effects of sensory adaptation to gaze direction (Lawson, Aylward, et al., 2017, Pellicano et al., 2013), as well as reduced effects of sensory adaptation in other perceptual domains (Lawson, Aylward, et al., 2015, Pellicano et al., 2007, Turi et al., 2015). Thus, we also simulated the effects of reducing normalisation with a reduced level of adaptation strength. This was done by setting the model parameter α to 50% of the value that best fit the data from Calder et al. (2008) (i.e., α = .35), to simulate 50% of typical adaptation effects. This is shown in Fig. 2B. As can be seen, when adaptation strength is reduced, the magnitude of perceptual aftereffects is reduced. However, the effect of varying normalisation on the profile of aftereffects across stimulus gaze directions remains qualitatively similar.

From the results of these simulations, we can draw qualitative predictions regarding how reduced normalisation will manifest in a sensory adaptation task. Firstly, when the encoded gaze direction is fully normalised to the summed activity across sensory channels, the predicted aftereffects show a characteristic profile across stimulus gaze directions. Specifically, peak aftereffects occur between the point of the adapter (−25°) and direct gaze (0°), with reduced aftereffects for test directions both more averted than the adapter (<−25°) and on the opposite side to the adapter (>0°). In contrast, when normalisation is reduced, the profile of predicted aftereffects differs such that peak aftereffects tend to occur to stimulus gaze directions that are at or beyond the point of the adapter (<−25°).

### 2.2 Participants

Participants were 27 adults with a diagnosis of ASD and 28 NT adults. These groups were closely matched in gender, age and IQ. Group demographics are shown in Table 1. ASD participants were recruited from the autism@icn database held by the University College London Institute of Cognitive Neuroscience. Participants with ASD had previously been diagnosed by an independent clinician, according to the DSM-IV (American Psychiatric Association, 2000) or ICD-10 criteria (World Health Organization, 2012). The Wechsler Adult Intelligence Scale (WAIS, third edition, UK) had previously been administered to assess IQ (Wechsler & Hsiao-pin, 2011). The Autism Diagnostic Observation Schedule (second edition) Module 4 (Lord et al., 2000) assessment was completed by a qualified administrator to assess symptom severity in the participants with ASD.

**Table 1:** Group demographics.

	Age	Gender (f:m)	ADOS	AQ	VIQ	PIQ	FSIQ
ASD	33.4 (9.2)	4:23	9.2 (3.5)	33.0 (9.4)	118.4 (14.0)	109.7 (14.3)	116.0 (13.7)
NT	31.3 (11.8)	9:19	n/a	14.2 (6.53)	117.5 (13.4)	112.7 (14.9)	116.4 (12.18)
Group difference	*t*(53) = .74, *p* = .46	χ^2^(1, *n* = 55) = 2.29, *p* = .13	n/a	*t*(52) = 8.58, *p* < .001	*t*(53) = .26, *p* = .80	*t*(53) = -.75, *p* = .46	*t*(53) = −.09, *p* = .93

Means and standard deviations are shown for the continuous measures. ADOS = Autism Diagnostic Observation Schedule, AQ = Autism Quotient (Baron-Cohen, Wheelwright, Skinner, Martin, & Clubley, 2001), VIQ = WAIS Verbal IQ, PIQ = WAIS Performance IQ, FSIQ = WAIS Full Scale IQ. One participant from the ASD group failed to complete the AQ.

All participants gave written informed consent to take part in this study and were financially compensated for their travel and time. This study was approved by the UCL Graduate School Ethics Committee (4357/002).

### 2.3 Face stimuli

**Fig. 3:**
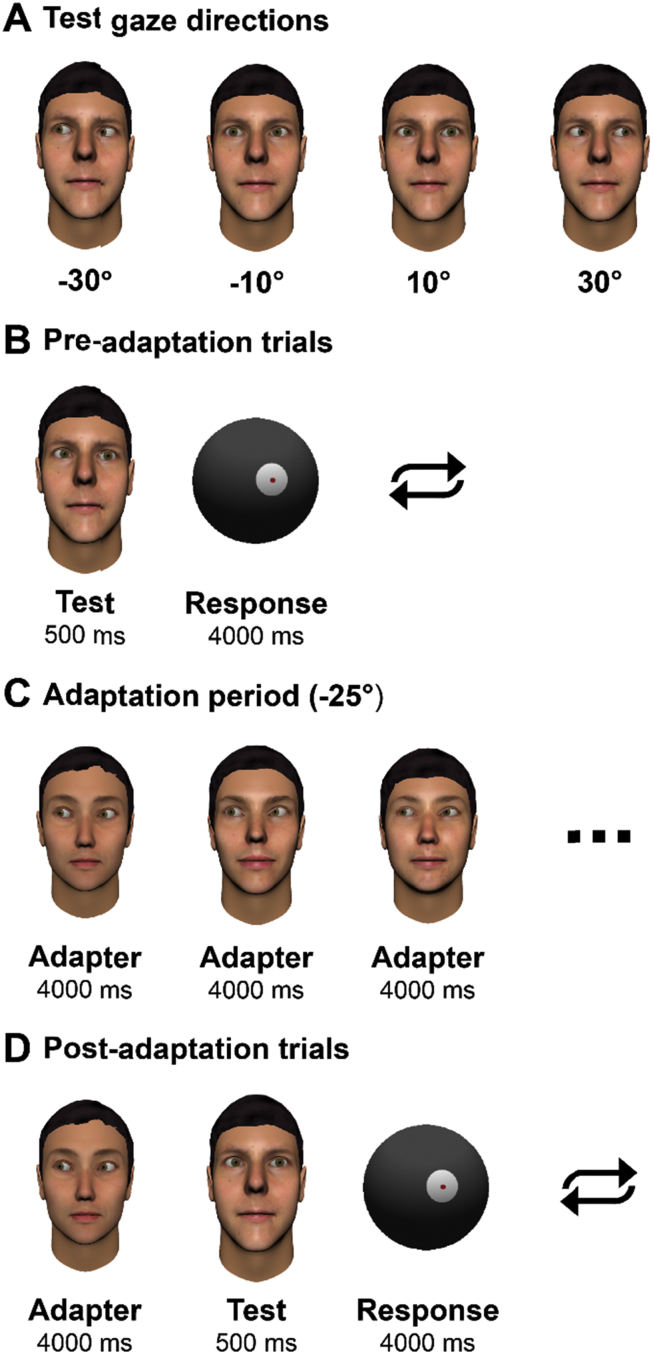
Task for measuring the psychophysical effects of sensory adaptation to gaze direction. **(A**–**B)** In a pre-adaptation test of gaze perception, participants were shown a series of faces with different horizontal directions of gaze, shown here for one of the six identities used. Participants indicated the direction in which each face was looking using a spherical pointer displayed on screen. **(C)** Participants were adapted to 25° averted gaze by viewing a series of faces with this direction of gaze for 4 sec each for a total of 1 min. **(D)** Following adaptation, participants were tested on their perception of the test stimuli again. In post-adaptation trials, participants were also shown ‘top-up’ adapter images to maintain adaptation throughout the task.

The stimuli were computer-generated images of faces, some examples of which are depicted in Fig. 3. Three-dimensional face models and textures were generated using FaceGen Modeller 3.5. We manipulated the rotation of the modelled eyes using Blender 2.70 before generating the 2d images shown to participants. This allowed precise control of the gaze direction relative to the viewer. Face images were generated for six identities, three male and three female. Left-right flipped versions of each image were also used to control for any asymmetries in the face models relevant to the horizontal dimension. The images were presented centrally on screen with an inter-ocular distance of 6.3 cm, which is approximately the human average (Fesharaki, Rezaei, Farrahi, Banihashem, & Jahanbkhshi, 2012).

### 2.4 Psychophysical task

Participants completed an adaptation task, modified from that described previously in Palmer and Clifford (2017a). This task is depicted in Fig. 3, and consists of (i) a pre-adaptation test of perceived gaze direction, (ii) an adaptation period in which participants were adapted to a particular direction of gaze, and (iii) a post-adaptation test of perceived gaze direction.

In the pre-adaptation period, participants were tested on their perception of faces with horizontal gaze deviation of 10° and 30° deviation leftwards and rightwards. This is a reduced set of test gaze directions compared to that we have examined previously (Palmer & Clifford, 2017a), but testing across this reduced set is sufficient to be diagnostic of the role of normalisation and adaptation in gaze processing, as indicated by the computational simulation presented in Section 2.1. Participants completed 12 trials for each test direction. Each test image was presented for 500 msec and followed by a spherical pointer that participants could rotate around the vertical axis between 90° leftwards and 90° rightwards using a mouse. Participants used this pointer to indicate the direction in which the face was looking. To keep the temporal structure of the experiment consistent across participants (i.e., not affected by their response times), there was a constant 4 sec period between onset of the response period and onset of the subsequent trial. If the participant did not respond within this period, the trial was repeated at the end of the block. Participants took 4–4.5 min to complete the pre-adaptation period.

The adaptation period was a 60-sec series of face images that all shared the same direction of gaze. This consisted of 3 face identities, shown in a random succession of 15 images presented for 4 sec each. Each participant was adapted on either 25° leftwards or 25° rightwards gaze, with the side of adaptation alternating between participants and balanced between the ASD and NT groups (with one extra rightwards-adapted participant in the NT group). Our previous results indicate that adaptation to leftwards versus rightwards gaze produces symmetrical effects on subsequent gaze perception (Palmer and Clifford, 2017a, Palmer and Clifford, 2017b). The data from those adapted to rightwards gaze was flipped such that it could be averaged and directly compared with data from those adapted to leftwards gaze.

Participants completed a simple detection task during the adaptation period to help maintain their attention to the face stimuli. Specifically, the iris colour of the eyes would occasionally switch from brown to blue for 200 msec. Participants were instructed to press a button as quickly as possible when this occurred. The iris colour change occurred at a random time in 20% of face images.

The post-adaptation test of gaze perception was identical to the pre-adaptation test, except that each trial began with a single ‘top-up’ adapter image displayed for 4 sec. This follows the design of previous gaze adaptation studies (e.g., Jenkins et al., 2006) and is intended to maintain adaptation throughout the task. Halfway through the post-adaptation test, participants were shown the adaptation period again to further ensure that the effects of adaptation did not dissipate during testing. Participants took 8–10 min to complete the post-adaptation period.

The presentation of adapter and test images was differed somewhat so that adaptation occurred to representations of gaze direction rather than to lower-level image properties, consistent with previous studies (Jenkins et al., 2006, Palmer and Clifford, 2017a). This included presenting the adapter images at 75% of the size of the test images, jittering the position of the test images randomly in each trial by up to 50 pixels in both the horizontal and vertical planes, and using different face identities for the adapter and test images such that participants were not tested on the same images that they were adapted on. There is compelling evidence that the effects of adaptation typically seen in this type of task are primarily due to adaptation of ‘abstract’ representations of gaze direction rather than adaptation to specific low-level image features (Palmer & Clifford, 2017b).

The task was presented on a Samsung SyncMaster (120 Hz) LCD monitor. Participants viewed the stimuli from approximately 50 cm, with their head position stabilised using a chin rest. Participants completed a practice task before each session to become acquainted with using the pointer method to report perceived gaze direction.

## 3 Results

### 3.1 Attention task

Participants performed well on the detection task, indicating that they were consistently attending to the faces during the adaptation period. A correct response was defined as when the participant pressed the button within 1 sec of the probe (iris colour change) appearing. Mean performance was 98% in the NT group and 99% in the ASD group. No individual missed more than one probe. There was a non-significant trend towards slightly slower RTs in ASD subjects (mean = 475 msec) compared to controls (mean = 443 msec), *t*(53) = −1.91, *p* = .062.

### 3.2 Testing qualitative predictions of the reduced normalisation model

**Fig. 4:**
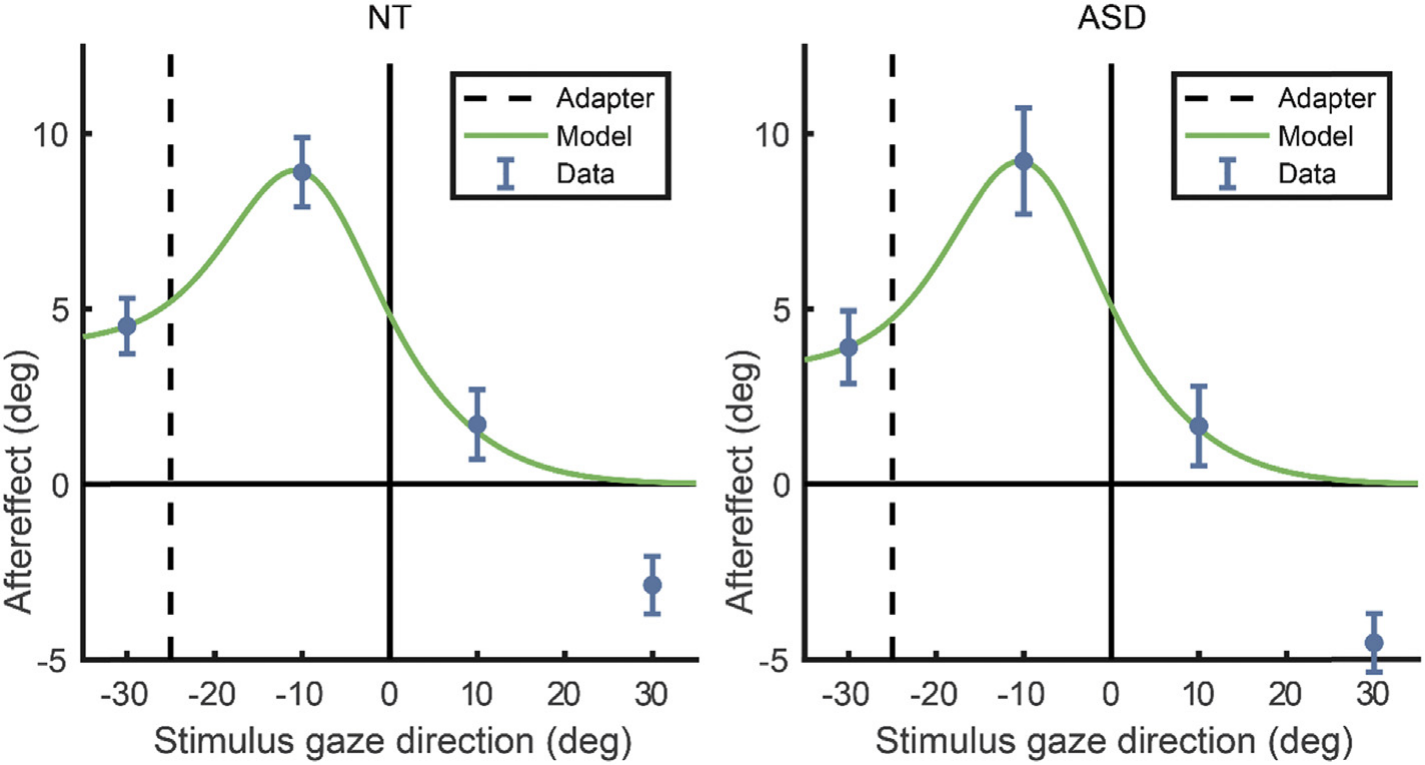
The effect of adaptation to 25° averted gaze on perceived gaze direction, averaged for NT and ASD groups. The model fits shown here are described in Section 4.

We saw above that reduced normalisation in the coding of gaze direction is expected to result in a different profile of perceptual aftereffects across test gaze directions. The profile of mean perceptual aftereffects for each group is displayed in Fig. 4. As can be seen, both groups show a difference in the magnitude of aftereffects across test gaze directions that is consistent with the predictions of the normalisation model. In particular, peak aftereffects occur between the point of the adapter (−25°) and direct gaze (0°), with reduced aftereffects for test directions both more averted than the adapter (−30°) and on the opposite side to the adapter (10° and 30°). It is apparent from these summary data that the groups show very similar effects of adaptation on perceived gaze direction.

To test formally whether the profile of perceptual aftereffects differed between adults with ASD and NT controls, we performed a 3-way mixed ANOVA on mean pointer responses, with Group (ASD *vs* NT) as a between-subjects factor, and Test Direction (−30°,-10°,10°,30°) and Block (pre-adaptation *vs* post-adaptation) as within-subjects factors.

There was a main effect of Block, *F*(1,53) = 69.44, *p* < .001, η^2^_p_ = .57, Test Direction, *F*(3,159) = 1094.91, *p* < .001, η^2^_p_ = .95, and a significant interaction between Test Direction and Block, *F*(3,159) = 50.35, *p* < .001, η^2^_p_ = .49. All other main effect and interaction terms were not significant (*p* > .05), thus there was no evidence for a difference between groups in the profile of perceptual aftereffects across test directions.

We used Bayesian statistics to quantify evidence in favour of the conclusion that there is no difference between ASD and NT controls in the effects of adaptation to gaze direction. A Bayesian mixed ANOVA on mean pointer responses was performed in JASP version 0.8.1.1 (JASP Team, 2018). As above, Group (ASD *vs* NT) was a between-subjects factor, and Test Direction (−30°,−10°,10°,30°) and Block (pre-adaptation *vs* post-adaptation) were within-subjects factors. For priors, the *r* scale fixed effects was .5 and the *r* scale random effects was 1. We report both BF_10_ values (quantifying evidence for the alternative hypothesis relative to the null) and BF_01_ values (quantifying evidence for the null hypothesis relative to the alternative) where appropriate. We draw on guidelines suggested by Jeffreys (1961) and Lee and Wagenmakers (2013) to interpret the strength of evidence.

The winning model in the Bayesian ANOVA included Block and Test Direction as factors, together with the interaction between these two factors, with decisive evidence for this model relative to the null model that included none of the factors or interaction terms, BF_10_ = 7.8 × 10^263^. Thus, the winning model did not include the interaction between Group, Block and Test Direction. Moreover, when Block, Test Direction, and their interaction were included in the null model, there was very little evidence for the alternative model that contained the interaction between Group, Block and Test Direction relative to this null, BF_10_ = 4.1 × 10^−4^. This corresponds to decisive support for the null, BF_01_ = 2.4 × 10^3^, thus providing evidence against the inclusion of this interaction term. In sum, these results provide strong support for there being no difference between ASD and NT groups in how the effects of adaptation differ across test directions.

### 3.3 Modelling group data

The model was first fit to the average data for each group. The channel sensitivities described in Section 1.1 were used together with a scaling factor that mapped the encoded gaze direction to the pointer response method used by participants in the experiment. The scaled multichannel response was as follows, where *S* is the scaling factor:

$$M_{S}\left(d\right)=\;M\left(d\right)*S.$$

A scaling factor was computed for each group. The sum of squared errors between the scaled multichannel response (when in the unadapted state) and the average pre-adaptation data across the group was minimised using the *fminsearch* function in MATLAB (R2017A). The best fitting scaling factors were very similar between the two groups: 40.77 for the NT group and 42.85 for the ASD group.

Next, the model described in Section 2.1. was fit to the participant aftereffect data, allowing the parameters describing the strength of adaptation (α) and the degree of normalisation (w) to vary. The normalisation parameter was constrained to between 0 and 1. The sum of squared errors was minimised between the average group aftereffects for each of the four test directions and the predicted aftereffects of the model for these same test directions. The model fit the data well in both groups, and the best-fitting parameters were very similar between groups, indicating a comparable degree of adaptation and normalisation (Fig. 4). To quantify model fits, we calculated the normalised residual variance by comparing the sum of squared aftereffects to the sum of squared error between the aftereffect data and the model, and report the variance accounted for by the model (ranging 0–100%). For the NT group, the model accounted for 92% of the variance, and the best-fitting parameters were α = .56 and w = .09. For the ASD group, the model accounted for 83% of the variance, and the best-fitting parameters were α = .56 and w = .07.

### 3.4 Modelling individual subjects

The model was also fit to individual participants to estimate the strength of adaptation and degree of normalisation on an individual level. This allowed us to test for group differences in these parameters statistically. The same procedure described in Section 3.3. was followed. Scaling factors were estimated for each participant by fitting the scaled multichannel response of the model (when in the unadapted state) to their individual pre-adaptation data. To find the best-fitting values of α andw, the sum of squared errors was minimised between the individual participant aftereffects for each of the four test directions and the predicted aftereffects of the model for these same test directions.

The best-fitting values for the model parameters are summarised for each group in Table 2. A one-sample *t*-test confirmed that the strength of adaptation (α) differed significantly from zero in both the NT group, *t*(27) = 12.04, *p* < .001, Hedges *g*_*rm*_ = 3.17, and the ASD group, *t*(26) = 8.76, *p* < .001, Hedges *g*_*rm*_ = 3.32. Similarly, a one-sample *t*-test confirmed that the normalisation parameter (w) differed significantly from 1 (where w = 1 corresponds to an absence of normalization) in both the NT group, *t*(27) = −14.29, *p* < .001, Hedges *g*_*rm*_ = 3.77, and the ASD group, *t*(26) = −10.14, *p* < .001, Hedges *g*_*rm*_ = 2.72. Thus, there was evidence for both adaptation effects and normalisation effects in both groups.

**Table 2:** Group differences in model parameters.

Best-fitting model parameters	NT		ASD				
	Mean	SD	Mean	SD	*t* (df)	*p*	*BF*_*01*_
α	.51	.22	.46	.28	.63 (53)	.53	2.19
w	.21	.29	.28	.37	−.78 (53)	.44	1.89
Scaling factor	40.77	8.35	42.85	7.46	−.98 (53)	.33	2.48
Variance explained (%)	68.44	22.14	60.46	29.57	1.14 (53)	.26	2.16

Independent samples *t*-tests indicated no significant differences in the model parameters between NT and ASD groups (*p* > .05, Table 2). Bayesian independent samples *t*-tests were also performed to quantify the evidence for the null hypothesis (that the strength of adaptation and normalisation did not differ between groups) relative to the alternative hypotheses that (i) adaptation would be reduced in the ASD group (i.e., α lower than in the NT group) and (ii) normalisation would be reduced in the ASD group (i.e., w higher than in the NT group). A Cauchy prior width of .7 was used in each case. These tests indicated ‘anecdotal’ or ‘weak’ evidence that the groups showed the same degree of adaptation and normalisation, i.e., BF_01_ = 1–3.

**Fig. 5:**
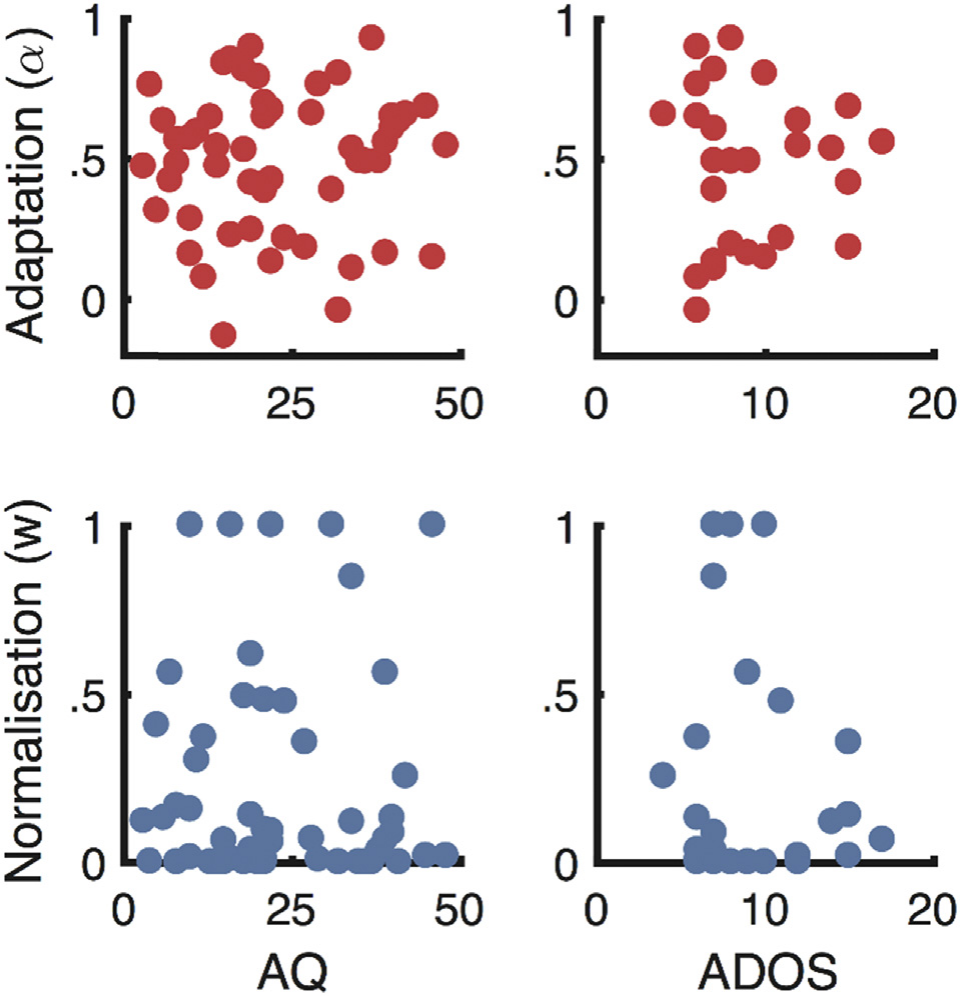
The relationship between autistic features and model parameters describing the strength of adaptation and normalisation. AQ data is from both ASD and NT participants, while ADOS data is from ASD participants only.

Similarly, Pearson's *r* indicated very small and non-significant linear correlations between the model parameters and autistic features (ADOS and AQ scores). Bayes' factors indicated substantial support for the null hypotheses of there being no linear relationship between these variables. A stretched beta prior width of 1 was used in computing these Bayes' factors. See Table 3 and Fig. 5.

**Table 3:** Correlations between model parameters and autistic features.

	AQ	ADOS
Adaptation (α)	*r* = .04 *p* = .78 BF_01_ = 5.67	*r* = −.002 *p* = .99 BF_01_ = 4.18
Normalisation (w)	*r* = −.007 *p* = .96 BF_01_ = 5.88	*r* = −.16 *p* = .43 BF_01_ = 3.12

## 4 Discussion

The data that we report here provide a clear picture of there being both typical *normalisation* and typical *adaptation* of sensory responses in adults with ASD, in the context of gaze processing. Perceptual aftereffects are typically considered to reflect the *population-coding* of the stimulus property across a set of stimulus-selective sensory channels (Suzuki, 2005). We have previously shown that for adaptation to averted gaze, the specific profile of perceptual aftereffects observed across stimulus gaze directions is indicative of several functional mechanisms, including the normalisation of sensory responses, adaptive habituation of channel-specific sensory gain, and the structure of the coding population (e.g., the number of sensory channels) (Palmer & Clifford, 2017a). The striking similarity between ASD and TD groups in their profile of perceptual aftereffects in the present study is therefore a strong testament to the underlying function of sensory coding in the gaze system in ASD. This coheres with previous work showing that individuals with ASD are able to report gross differences in where a face is looking when asked to (Leekam et al., 1997, Wallace et al., 2006), and exhibit similar perceptual biases regarding others' gaze direction to those found in NT individuals (Pell et al., 2016). However, unusual behavioural and physiological responses to others' gaze are a hallmark feature of ASD, both during infanthood and later life (Lai et al., 2014, Senju, 2013). It is perhaps surprising, therefore, that the sensory coding of gaze direction appears to occur in such a typical manner, suggesting that the differences in response to others' gaze in ASD relate to function at a higher level in the system, such as the interpretation of gaze direction within the social context or the spontaneous following of others' gaze (e.g., Senju, Southgate, White, & Frith, 2009).

The evidence that we report here for robust normalisation of sensory responses in adults with ASD conflicts with the proposal that this condition is characterised by a widespread reduction in the normalisation of sensory responses (Rosenberg et al., 2015). In introducing this hypothesis, Rosenberg and colleagues demonstrated that a simulation of reduced normalisation in early visual processing (V1) predicts low-level psychophysical differences that are similar to certain findings in ASD, namely in the context of spatial suppression (Foss-Feig, Tadin, Schauder, & Cascio, 2013) and the spatial gradient of facilitatory effects produced by visual attention (Robertson, Kravitz, Freyberg, Baron-Cohen, & Baker, 2013). Here we find that a psychophysical signature of normalisation in gaze processing is as robustly apparent in individuals with ASD as in controls. One potential explanation for this conflict in findings is the contrasting levels of visual function examined: it may be that normalisation is reduced in ASD in very early visual processing (e.g., V1), but intact in higher-level visual processing (e.g., anterior STS, which is implicated in the adaptive coding of gaze direction that we examine in the present study; Calder et al., 2007). This would suggest that the theory of reduced normalisation in ASD is less one of systemic differences in neural computations, but rather a more circumscribed account of low-level visual characteristics. An alternative possibility is that local divisive normalisation computations are preserved across the brain in ASD (i.e., both in gaze processing and in low-level visual processing); further empirical research that supplements Rosenberg and colleague's simulation analyses by more directly testing the notion of reduced normalisation in low-level visual responses in ASD will thus be valuable to the field.

The present results may also be helpful in adjudicating between the type of divisive computations that are potentially compromised in ASD. A recent computational approach to ASD modelled the learning of environmental contingencies in terms of hierarchical Bayesian inference, in which new sensory data is flexibly weighted in accordance with learnt estimates of multiple forms of sensory and environmental uncertainty (Lawson, Mathys, & Rees, 2017). This study finds evidence for differences in ASD in the context-dependent weighting of sensory information, reflected also in phasic pupil dilation, implicating long-range noradrenergic neuromodulation of cortical responses. Taking these previous findings together with the results of the present study, it may be that *local* divisive computations are preserved in ASD (e.g., reflecting inhibitory interactions between neighbouring pools of cells that together code for perceived gaze direction), while more *distant* modulation of sensory responses may be compromised, such as in the distributed cortical gain control thought to be implemented by noradrenergic activity originating in the locus coeruleus (for discussion of the latter, see Lawson, Friston, et al., 2015, Lawson et al., 2014).

In the present study, we observed strong sensory aftereffects in adults with ASD following adaptation to averted gaze. This conflicts with previous findings of reduced effects of adaptation to gaze direction in children with ASD (Pellicano et al., 2013) and adults with ASD (Lawson, Aylward, et al., 2017). There is also evidence for reduced adaptation in ASD to other higher-level visual features, such as face identity (Pellicano et al., 2007) and numerosity (Turi et al., 2015), and to lower-level sensory properties such as loudness (Lawson, Aylward, et al., 2015). A key difference between the method of the present study and past studies of adaptation to gaze direction in ASD is the use of a continuous rather than categorical measure of perceived gaze direction. Specifically, in the present study, participants indicated the direction in which each face was looking by setting the rotation of a pointer, while in the previous two studies of adaptation to gaze direction in ASD, participants categorised whether the face was looking directly towards them or away from them. The difference between studies may therefore reflect a difference between groups in how gaze directions are categorised. For instance, in both previous studies, participants with ASD more commonly categorised gaze as direct at baseline (i.e., pre-adaptation) compared to NT controls (Lawson, Aylward, et al., 2017, Pellicano et al., 2013), suggesting a wider ‘cone of direct gaze’ (i.e., the range of gaze deviations that are typically classified as looking direct), or greater ambiguity in the boundaries between direct and averted gaze. The reduced effects of adaptation in individuals with ASD observed in these previous studies might then reflect a difference in how adaptation interacts with the categorisation of gaze direction, rather than a difference between groups in the lower-level effects of adaptation on the sensory coding of gaze direction.

On a more technical note, the present data indicate strong sensory aftereffects following adaptation to averted gaze for stimulus test directions that are averted on the same side of the adapter (i.e., at −10° and −30° in Fig. 4), but also an aftereffect for test stimuli that are highly averted on the opposite side of the adapter (i.e., at 30° in Fig. 4). In both cases, the perceived gaze direction is drawn towards direct gaze. The aftereffects produced on the opposite side to the adapter are not accounted for by the model of gaze perception that we employ here, and might reflect, for example, the existence of additional adaptive mechanisms that encode averted gaze independent of side (Perrett et al., 1985). We note, however, that these aftereffects on the opposite side to the adapter are apparent in both the NT and ASD groups.

It is worth noting that the individuals with ASD tested here were ‘high-functioning’ in the sense that IQ scores were not impaired relative to the general population (the mean FSIQ was 116 in both ASD and NT groups). However, sensory and neural differences in ASD are regularly studied (and observed) in adults with unimpaired IQ. One pertinent example is a recent study that observed reduced effects of adaptation to gaze direction in adults with ASD, with a very similar sample (mean FSIQ = 115) (Lawson, Aylward, et al., 2017). Similarly, the simulations of reduced normalisation in low-level visual processing presented in Rosenberg et al. (2015) are based substantially on data collected from clinical samples with above-average IQ (e.g., Foss-Feig et al., 2013, FSIQ = 116; Robertson et al., 2013, non-verbal IQ = 114).

Unusual responsiveness to others' gaze is a hallmark feature of ASD during both childhood and adulthood; however, the present data indicate that key functional processes involved in the population coding of gaze direction, namely the normalisation and adaptation of neural responses, occur in a typical manner in adults with this diagnosis. This conflicts with the recent proposal that ASD is characterised by a widespread failure of divisive computations across the brain (Rosenberg et al., 2015). When taken together with recent evidence for altered noradrenergic regulation of how sensory information is context-dependently weighted in updating expectations about environmental contingencies (Lawson, Mathys, et al., 2017), a view emerges that divisive computations that occur locally within functional regions may be intact in ASD, while more distal gain control may be compromised.

## Competing interests

The authors have no competing interests to declare.
